# The Prevalence of Nomophobia and Its Impact on Academic Performance of Medical Undergraduates at the College of Medicine, Umm Al-Qura University, Makkah City, Saudi Arabia

**DOI:** 10.7759/cureus.51052

**Published:** 2023-12-24

**Authors:** Safa H Alkalash, Abdullah K Aldawsari, Salman S Alfahmi, Abdulaziz O Babukur, Rudhab A Alrizqi, Khalid F Salaemae, Raghad O Al-Masoudi, Khalid A Basamih

**Affiliations:** 1 Community Medicine and Health Care, Umm Al-Qura University, Al-Qunfudah, SAU; 2 Family Medicine, Menoufia University, Shebin Elkom, EGY; 3 Medical College, Umm Al-Qura University, Makkah, SAU; 4 Medical College, Umm Al-Qura University, Al-Qunfudah, SAU

**Keywords:** mental health, nomophobia, mobile phone, medical undergraduates, academic performance

## Abstract

Background: Nomophobia, or no mobile phone phobia, is a term used to describe psychological conditions when people fear being without a mobile phone.

Objectives: This study aimed to measure the prevalence of nomophobia, its associated factors, and its impact on the academic performance of medical undergraduates at Umm Al-Qura University (UQU), Makkah City, Saudi Arabia.

Methods: A descriptive cross-sectional study included a simple random sample of 595 medical undergraduates. The study administered the Nomophobia Questionnaire, which had been validated in previous articles, using an interview-based model. The collected data were reviewed and entered into the IBM SPSS Statistics for Windows, Version 26 (Released 2019; IBM Corp., Armonk, New York) for analysis.

Results: A total of 595 medical students were included in this study. Most of them (99%, n = 589) had different degrees of nomophobia, while only a small percentage (1%, n = 6) did not exhibit it. The younger medical students (18-19 years old) showed significantly higher percentages of moderate to severe nomophobia in comparison to older students (P = 0.028), while there was no significant relationship between nomophobia and academic performance, despite the fact that 128 (77.1%) of students with severe nomophobia had grade point averages (GPAs) of 3.5-4.0, compared to 244 (69.1%) of students with moderate nomophobia (P = 0.150).

Conclusions: This study concludes that the prevalence rate of nomophobia is generally high among medical undergraduates at UQU in Makkah City, Saudi Arabia. This disorder was detected more among younger medical undergraduates, with no significant effects on their academic performance. It is mandatory to conduct more studies with larger sample sizes of different university students (medical and non-medical) to identify factors that lead to the development of nomophobia. Qualitative research among groups of university students will add more deep information about this disorder. Health education programs should be designed and targeted at university students, focusing on the harmful health effects of excessive use of mobile phones and measures to prevent this disorder.

## Introduction

Technology is an essential component of people's lives. The mobile phone is a technological product that makes human lives more accessible and flexible, becoming an indispensable tool. Moreover, the mobile phone is the first site to answer any question and to connect to the world [1]. Users of smartphone technology state that it has become an extension of their body, determining their identity and way of being [1]. However, as the demand for mobile phones has grown, addiction has led to the development of a psychological condition known as nomophobia [1].

The fourth edition of the Diagnostic and Statistical Manual of Mental Disorders (DSM-4) defined nomophobia as a 'phobia for a particular/specific thing' [2]. Therefore, nomophobia, or no mobile phone phobia, is a term used to describe psychological conditions when people fear being without a mobile phone. Individuals who develop nomophobia show signs and symptoms like anxiety, respiratory alterations, trembling, perspiration, agitation, disorientation, and tachycardia. Moreover, nomophobia shares signs and symptoms with other mental disorders. That is why it is diagnosed by exclusion [3]. Furthermore, prolonged usage of a cell phone is linked to negative consequences on one's physical health, including migraines, numbness from continuous use of a cell phone, and injuries from repetitive motion in the back, shoulders, elbows, and fingers [3]. Although the fifth edition of the Diagnostic and Statistical Manual of Mental Disorders (DSM-5) formally recognizes gambling disorder as the first behavioral addiction disease and includes it in the updated chapter of Substance-Related and Addictive Disorders [4], it does not include nomophobia disorder. However, the psychopathological consequences of cell phone use warrant much more attention [2].

Nomophobia is more prevalent among young adults and teenagers [5]. Many studies have revealed a higher prevalence rate of nomophobia among university students in different countries around the world, such as India, South America, Saudi Arabia, and Indonesia [6-10]. The main drawbacks of nomophobia among students are sleep disturbances and poor academic achievement. A study done in India in 2020 shows that addiction to mobile phones reflects poor academic performance for school and college students [6]. Nomophobia affects not only the academic performance of students but also their lives with anxiety, depression, high stress levels, low physical activity, and a higher body mass index (BMI) [7, 9]. Medical students have a special situation as they will be physicians and doctors in the near future. Nomophobia may negatively affect their communication skills with others. An Egyptian study found that nomophobia affected all medical residents with different specialties, and about half of the residents in this study who had poor doctor-patient relationships had severe nomophobia [11]. There are no adequate studies among Umm Al-Qura University (UQU) medical students that describe nomophobia. Therefore, this study aimed to measure the prevalence of nomophobia among medical undergraduates and its impact on their academic performance at UQU, Makkah City, Saudi Arabia.

## Materials and methods

Study design

A descriptive cross-sectional study design was chosen to determine the prevalence of nomophobia among medical undergraduates at UQU in Makkah City and its effects on their academic performance.

Study setting

The study was carried out at the College of Medicine at UQU, Makkah City, Saudi Arabia, among medical undergraduates during the academic year 2022-2023.

Sample size calculation

The Raosoft sample size calculator was utilized to estimate the minimum sample size required for this study, considering the following: the population size of medical students at UQU is about 1,362 students, with a confidence interval (CI) level set at 95%, an anticipated frequency of 50%, and a design effect of 1. Accordingly, the minimum sample size required is 340 participants.

Study population

The study included 595 students based on the inclusion criteria. Data were collected through a simple random sampling technique with the targeted population. The study's inclusion criteria encompassed all medical students in all grades, from the first academic year to the level of medical internship, of both genders, and excluded those who had previously been diagnosed with any psychiatric disorder, such as anxiety, depression, or bipolar disorder. Participants who did not give informed consent for the study or who did not meet the participation criteria were also excluded.

Data collection

Data were collected from April to June 2023. All participants gave informed consent prior to completing the research questionnaire. An English questionnaire was administered and completed voluntarily by the participants using an interview-based model. The applied questionnaire was simple, succinct, and easy to understand for medical students and was validated [12]. It was distributed electronically via data collectors to the medical students at UQU in Makkah City. The questionnaire took two to five minutes to complete.

The questionnaire was composed of four sections. Section one included an informed consent form. Section two consisted of sociodemographic data and included seven demographic questions asking about age, gender, marital status, and residency. It also contained three questions that assessed psychological disorders. Section three, for academic information, included the academic year and their grade point averages (GPAs). Section four had twenty multiple-choice questions to assess the prevalence of nomophobia among medical undergraduates and its severity.

Statistical analysis

The data were collected, reviewed, and then fed into the IBM SPSS Statistics for Windows, Version 26 (Released 2019; IBM Corp., Armonk, New York). Descriptive analysis was conducted by prescribing frequency distribution and percentage for study variables, including students' personal data, residence area, academic years, and GPAs. Additionally, students' mean scores with standard deviations (mean ± SD) for different nomophobia items were tabulated, while their overall nomophobia level and its distribution with GPA were graphed. The overall nomophobia score of the Nomophobia Questionnaire (NMP-Q) was calculated by summing responses to each item. Higher scores indicate more severe levels of nomophobia. The overall score was categorized as absent (score = 20), mild (21 ≤ NMP-Q Score < 60), moderate (60 ≤ NMP-Q Score < 100), and severe (100 ≤ NMP-Q Score ≤ 140) [12]. Cross-tabulation for showing the relation between nomophobia and medical students' personal data, and the relation between nomophobia and medical students' academic performance, was carried out with the Pearson chi-squared test; if the frequency distributions were small, an exact probability test was applied. With an alpha threshold of 0.05, all statistical techniques were two-tailed, and a P value of less than or equal to 0.05 was deemed significant.

Ethical considerations

The research adhered to the World Medical Association's Helsinki Declaration (1964), most recently revised in 2008. Before answering the study questionnaire, all participants were asked to give informed consent for the study, ensuring that all data would be processed anonymously. Identifying information was not collected from participants; no private information was collected, and all responses were kept confidential.

## Results

A total of 595 medical students completed the survey. Students' ages ranged from 18 to 28 years, with a mean age of 21.1 ± 2.0 years. A total of 350 (58.8%) students were female, and 582 (97.8%) were single. Regarding the academic year, 365 (61.3%) students were in the basic years (1st to 3rd), while 202 (33.9%) were in the clinical years (4th to 6th), and 28 (4.7%) were interns. Regarding GPA, it was 3.5-4.0 among 422 (70.9%) students and 2.75-3.49 among 151 (25.4%) (Table 1).

**Table 1:** Personal characteristics of medical undergraduates

Personal data	N	%
Age in years		
18-19	162	27.2%
20-21	199	33.4%
22-23	150	25.2%
24+	84	14.1%
Mean ± SD (21.1 ± 2.0)		
Gender		
Male	245	41.2%
Female	350	58.8%
Marital status		
Single	582	97.8%
Married	11	1.8%
Divorced/widow	2	0.3%
Academic year		
First	147	24.7%
Second	92	15.5%
Third	126	21.2%
Fourth	61	10.3%
Fifth	81	13.6%
Sixth	60	10.1%
Medical internship	28	4.7%
Academic phase		
Pre-clinical	365	61.3%
Clinical	202	33.9%
Internship	28	4.7%
Grade point average (GPA)		
1.75-2.74	22	3.7%
2.75-3.49	151	25.4%
3.5-4.0	422	70.9%

Descriptive analysis of nomophobia questionnaire items among medical students at UQU, Makkah City, Saudi Arabia, revealed that the highest mean score was for being annoyed when they could not look up information on their smartphone when they wanted to do so (5 out of 7), followed by being annoyed when they were unable to use their smartphone and/or its capabilities when they wanted to do so (4.9 out of 7), feeling uncomfortable without constant access to information through their smartphone (4.8 out of 7), not being able to check their smartphone for a while, and feeling the desire to check it (4.8 out of 7). If he or she did not have a smartphone with him or her, he or she would be worried because family and/or friends could not reach him or her (4.8 out of 7). If he or she did not have a data signal or could not connect to Wi-Fi, he or she would constantly check to see if he or she had a signal or could find a Wi-Fi network (4.6 out of 7). If he/she did not have a smartphone, he/she would feel anxious because he/she could not instantly communicate with his/her family and/or friends (4.5 out of 7), he/she could not keep in touch with his/her family and/or friends (4.5 out of 7), and finally, he/she would feel anxious because he/she could not check his/her email messages (3.5 out of 7) (Table 2).

**Table 2:** Descriptive of nomophobia questionnaire items among medical undergraduates

Nomophobia questionnaire items	Mean	±SD
I would feel uncomfortable without constant access to information through my smartphone.	4.8	1.8
I would be annoyed if I could not look information up on my smartphone when I wanted to do so.	5.0	1.7
Being unable to get the news (e.g., happenings, weather, etc.) on my smartphone would make me nervous.	3.8	1.8
I would be annoyed if I could not use my smartphone and/or its capabilities when I wanted to do so.	4.9	1.7
Running out of battery in my smartphone would scare me.	3.9	1.9
If I were to run out of credits or hit my monthly data limit, I would panic.	3.9	1.8
If I did not have a data signal or could not connect to Wi-Fi, then I would constantly check to see if I had a signal or could find a Wi-Fi network	4.6	1.8
If I could not use my smartphone, I would be afraid of getting stranded somewhere.	4.3	1.7
If I could not check my smartphone for a while, I would feel a desire to check it.	4.8	1.8
If I did not have my smartphone with me, I would feel anxious because I could not instantly communicate with my family and/or friends.	4.5	1.8
If I did not have my smartphone with me, I would be worried because my family and/or friends could not reach me.	4.8	1.8
If I did not have my smartphone with me, I would feel nervous because I would not be able to receive text messages and calls	4.4	1.8
If I did not have my smartphone with me, I would be anxious because I could not keep in touch with my family and/or friends.	4.5	1.8
If I did not have my smartphone with me, I would be nervous because I could not know if someone had tried to get a hold of me.	4.4	1.8
If I did not have my smartphone with me, I would feel anxious because my constant connection to my family and friends would be broken.	4.2	1.8
If I did not have my smartphone with me, I would be nervous because I would be disconnected from my online identity.	3.6	1.9
If I did not have my smartphone with me, I would be uncomfortable because I could not stay up to date with social media and online networks.	3.9	1.9

**Figure 1:**
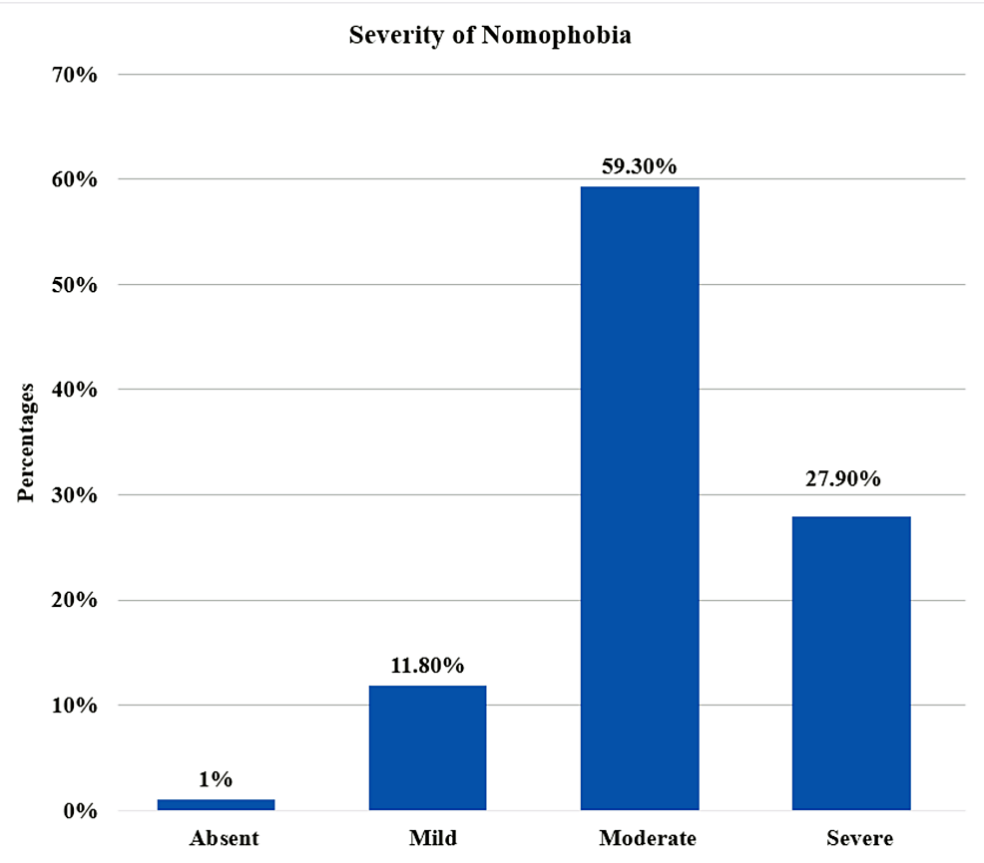
Prevalence of nomophobia among medical students at Umm Al-Qura University, Makkah City, Saudi Arabia

The prevalence of nomophobia among medical undergraduates at UQU, Makkah City, Saudi Arabia, was 99% (n = 589); moderate-severity nomophobia was predominant, representing 59.3% (n = 353); and severe form constituted 27.9% (n = 166) (Figure 1).

Regarding the relationship between nomophobia and medical students' personal data, precisely 95 (63.3%) students aged 22-23 exhibited moderate nomophobia, with recorded statistical significance (P = 0.028). The moderate degree of nomophobia is significantly higher among medical undergraduates in their pre-clinical phase (P = 0.001) (Table 3).

**Table 3:** Relation between nomophobia and medical undergraduates' personal data *The P-value has been calculated using the chi-squared test. Statistical significance is at the P-value less than 0.05. ^$^Fisher's exact test.

Personal data	Nomophobia level						P-value^*^
	Absent/Mild		Moderate		Severe		
	N	%	N	%	N	%	
Age in years							0.028
18-19	14	8.6%	99	61.1%	49	30.2%	
20-21	23	11.6%	121	60.8%	55	27.6%	
22-23	20	13.3%	95	63.3%	35	23.3%	
24+	19	22.6%	38	45.2%	27	32.1%	
Gender							0.551
Male	34	13.9%	148	60.4%	63	25.7%	
Female	42	12.0%	205	58.6%	103	29.4%	
Marital status							0.383^$^
Single	74	12.7%	347	59.6%	161	27.7%	
Married	1	9.1%	6	54.5%	4	36.4%	
Divorced/widow	1	50.0%	0	0.0%	1	50.0%	
Academic phase							0.001
Pre-clinical	33	9.0%	227	62.2%	105	28.8%	
Clinical	37	18.3%	117	57.9%	48	23.8%	
Internship	6	21.4%	9	32.1%	13	46.4%	

On studying the relationship between nomophobia and medical students' academic performance, it was found that exactly 128 (77.1%) students with severe nomophobia had GPAs of 3.5-4.0, with a non-significant difference from other students with mild and moderate degrees of nomophobia (P =0.150) (Table 4).

**Table 4:** Relation between nomophobia and medical undergraduate's academic performance *The P-value has been calculated using the chi-squared test. Statistical significance is at the P-value less than 0.05 level.

GPA	Nomophobia level						P-value
	Absent/mild		Moderate		Severe		
	N	%	N	%	N	%	
1.75-2.74	5	6.6%	11	3.1%	6	3.6%	0.150
2.75-3.49	21	27.6%	98	27.8%	32	19.3%	
3.5-4.0	50	65.8%	244	69.1%	128	77.1%	

**Figure 2:**
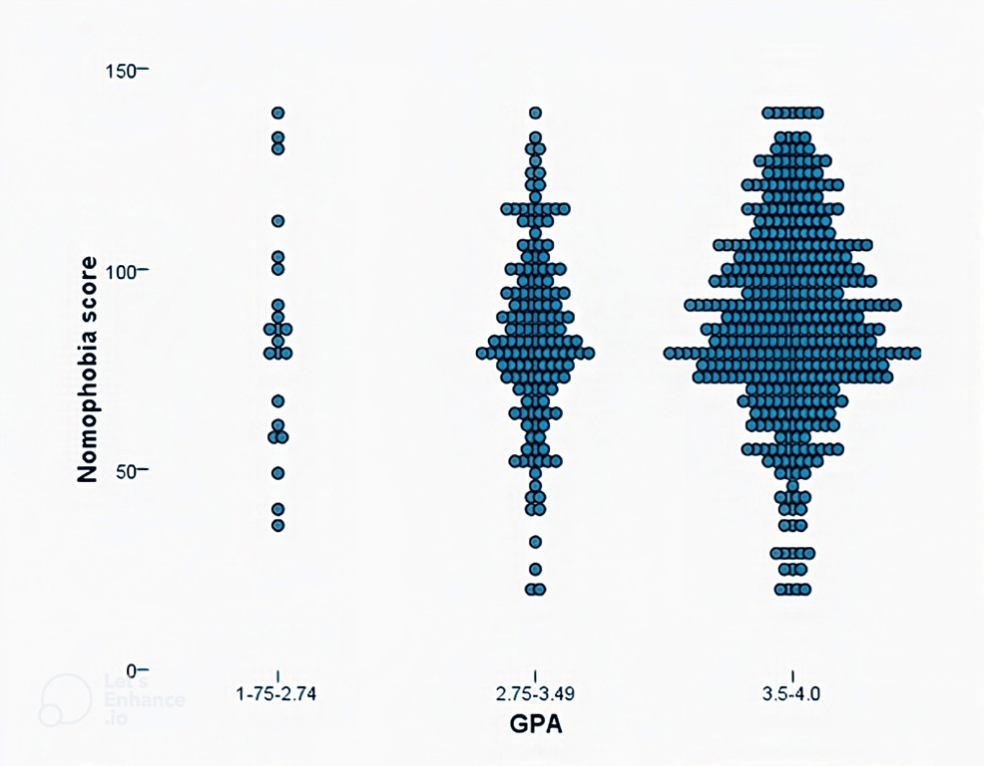
Distribution of nomophobia score among medical undergraduates by their GPAs

When examining the distribution of nomophobia scores among medical students by their GPAs, it is clear that most cases had GPAs of 3.5-4.0 points, while the lowest cases had GPAs of 1.7-2.74 points, with no statistically significant difference regarding the distribution level (Figure 2).

## Discussion

The current study revealed that the prevalence of nomophobia among medical undergraduates at UQU, Makkah City, Saudi Arabia, was 99% (n = 589), which is consistent with the findings of studies conducted in India, the United States, Oman, and Saudi Arabia [13-16]. The studies consistently report a high prevalence of nomophobia among students, ranging from 85.3% to 99.8%. Specifically, the research by Sethia et al. [13] in India found that all participants exhibited some degree of nomophobia, while only one did not suffer from it. Similarly, Cain and Malcom [14] found a prevalence of 99.5% among students in the United States, while a study in Oman reported a prevalence of 99.33% [15]. The most recent study from Saudi Arabia found a prevalence of 85.3% among university students [16]. The high prevalence rate of nomophobia among medical students is a problem that requires greater attention. Medical students, in particular, can be prone to nomophobia because they rely on their cell phones to meet the demands of academics and medical college. On the other hand, two studies done in India found that only 25.21% of students have nomophobia [17], and the other showed a prevalence of 50.70% and 30.26% have moderate and severe nomophobia [18]. Differences in the study population and data collection tools might contribute to the discrepancy in prevalence among different studies.

The result of this study indicates that nomophobia and age have a statistically significant relationship, and the younger generation is most affected by nomophobia. This finding agrees with other studies, showing an increase in the prevalence of nomophobia disorder among young people [19-20]. A possible explanation for this result may be that the medical students in Makkah City like to remain at home and use smartphones because there are fewer social activities and entertainment opportunities.

The current study's result shows no significant association between gender and nomophobia. This finding corroborates the studies of medical students in Saudi Arabia and India, which suggests that this disorder is equally prevalent among the study group irrespective of gender [21-22]. While the findings of other studies do not support the current study, they do suggest that males are more prone to nomophobia than females [23-24], and the result of a survey among dental students in India showed that the prevalence of females is more elevated than that of males [17]. It seems possible that these results are due to the sample being collected from only one medical college and the small sample size, which may not reflect the generalized scenario.

The present research revealed that medical students in the clinical phase showed a lower percentage of nomophobia compared to pre-clinical phase students, which is consistent with the findings of previous studies [7,25]. This observation may be attributed to the excessive use of social media and newer technologies by young individuals. Furthermore, younger students tend to be more knowledgeable about the latest technological devices, websites, and applications, which may contribute to their increased risk of nomophobia [26]. The findings of the present survey support the notion that senior students may have developed a better ability to manage their technology use. These findings could be due to their exposure to the clinical setting and the demands of their medical curriculum, which may have prioritized more practical and hands-on activities over recreational technology use. However, further research is needed to explore the potential factors that contribute to the high prevalence of nomophobia among medical undergraduates in their pre-clinical phase.

In this study, approximately 128 (77.1%) medical students with severe nomophobia had GPAs of 3.5-4.0, with a non-significant difference from other students with mild and moderate nomophobia (P = 0.150). This finding is similar to previous studies in Oman and Saudi Arabia [15-16]. While another Saudi study in Riyadh detected a significant correlation between nomophobia and poor academic achievement among university students attending King Saud University (KSU) [21], the variability between the current study and the old one may be due to differences in both study settings, as the other Saudi study was conducted in Riyadh city, which is a big central city with more urbanization characteristics than Makkah City. Additionally, the previous research included a large sample of 2367 students from varied colleges at KSU, while this study involved only medical students at UQU.

Limitations and strengths

The current study on nomophobia behavior among medical students has limitations, including that the sample was collected from only one medical college in Saudi Arabia, and the findings from the study cannot be generalized. Therefore, to gain more insights into the implications of nomophobia among students, future studies with larger sample sizes and more diverse student populations are recommended. Moreover, mental health assessments should be included in such studies, particularly among those with nomophobia. Despite the previously mentioned shortages, this study on nomophobia among UQU medical students sheds light on the potential negative consequences of smartphone addiction, revealing a high prevalence of nomophobia among medical undergraduates. The study's specific focus on nomophobia allowed for a more detailed exploration of the topic, with reliable and valid methods such as questionnaires and interviews employed to collect data. Consequently, these methods provided an accurate understanding of the prevalence of nomophobia and its related factors among medical undergraduates, as well as its effects on their educational performance.

## Conclusions

In this study, the prevalence rate of nomophobia was found to be high among medical undergraduates at UQU. This disorder appears to be getting more common among younger medical students than older ones, without affecting their academic achievement. Based on the findings, we recommend employing more studies among a wide scale of university students searching for prevalence, risk factors, and negative effects of nomophobia on health, lifestyle, and the educational process. Qualitative research designs are recommended to understand the full perspective of this disorder better. Furthermore, research should be implemented to perform a full psychological assessment for those with nomophobia. Finally, health education programs should be done, targeting university students, particularly younger students, to promote proper and effective mobile phone use among them and minimize any adverse impacts.
